# Construction of miniantibodies for the in vivo study of human autoimmune diseases in animal models

**DOI:** 10.1186/1472-6750-7-46

**Published:** 2007-08-01

**Authors:** Roberto Di Niro, Federica Ziller, Fiorella Florian, Sergio Crovella, Marco Stebel, Marco Bestagno, Oscar Burrone, Andrew RM Bradbury, Paola Secco, Roberto Marzari, Daniele Sblattero

**Affiliations:** 1 Department of Biology, University of Trieste, 34127 Trieste, Italy; 2 Department of Reproductive and Development Science, University of Trieste, 34127 Trieste, Italy; 3 CSPA, University of Trieste, 34127 Trieste, Italy; 4 International Centre for Genetic Engineering and Biotechnology, 34012 Trieste, Italy; 5 Bioscience Division, Los Alamos National Laboratory, 87545 Los Alamos, NM, USA; 6 Department of Medical Sciences, IRCAD, University of Eastern Piedmont, 28100 Novara, Italy

## Abstract

**Background:**

Phage display antibody libraries have been made from the lymphocytes of patients suffering from autoimmune diseases in which the antibodies are known to play a role in the pathogenesis or are important for the diagnosis of the disease. In the case of Celiac Disease, the immune response is directed against the autoantigen tissue transglutaminase. However, despite numerous studies, the role of these antibodies in the pathogenesis of this disease has not been elucidated.

**Results:**

We were able to engineer specific anti-transglutaminase antibody fragments in the form called "miniantibody". These are produced by genetic fusion of anti-tTG scFv to Human, Mouse or Rat Fc domains, making them suitable for in vivo expression. The results obtained here indicate that the miniantibody molecule is efficiently secreted, and that the reactivity to the antigen is retained even after fusion to heterologous Fc domains. Further analysis demonstrate that the molecule is secreted as homodimeric, mimicking original antibody structure. Finally, the in vivo expression in mice leads to detectable serum levels with no apparent gross immune response by the host.

**Conclusion:**

In this work we demonstrated the usefulness of a method for the in vivo expression of miniantibodies specific to transglutaminase, corresponding to the autoimmune specificity of Celiac Disease. This can be proposed as a general method to study the pathogenic role of autoimmune antibodies in autoimmune diseases.

## Background

Autoimmunity is an important cause of disease in humans, it is estimated to affect at least 3% to 5% of the human population and depends on a failure of the mechanisms normally responsible for maintaining self-tolerance (for a review see [1]). Although many factors causing these diseases, including the genes that may predispose to autoimmunity, have been identified, the aetiology of most autoimmune diseases remains obscure. Much interest has focused on the analysis of the immune factors leading to the tissue lesions. In some cases the cellular immune response stimulated by lymphokines seems to play a major role, whereas in others the humoral antibody response is deemed prevalent. Functional genomics may offer a solution to these problems by using biological systems which allow the massive interaction between an autoimmune patient's cloned antibody repertoire and individual antigens. One of these systems is phage display, a technique which involves the coupling of phenotype to genotype in a selectable format. It has been extensively used in molecular biology to study protein-protein interactions and one of the most successful applications of phage display has been the isolation of monoclonal antibodies to purified antigens [2-5]. In addition to libraries from naive or immunized sources, phage antibody libraries have also been made from patients suffering from autoimmune diseases. This work has been most extensively carried out with thyroid disease [6], systemic lupus erythematosus [7], paraneoplastic encephalomyelitis [8], myasthenia gravis [9] and type 1 diabetes mellitus [10]. In a recent work we described the antibody response in Celiac Disease (CD) [11]. This is a genetic illness strongly linked to HLA DQ2, characterized by flattening of the intestinal mucosa and malabsorption. The pathogenesis is precipitated by dietary exposure to wheat gluten and similar proteins in rye, barley and possibly oats [12]. The disease is characterized by the presence of specific antibodies recognizing gliadins, food proteins and an endomysial autoantigen, identified as tissue transglutaminase (tTG) [13]. We recently made and selected phage antibody libraries from the DNA isolated from CD patient lymphocytes and were able to isolate single-chain antibody fragments (scFv) to tTG showing their specific production by intestinal lymphocytes, indicating that the site of synthesis of these antibodies is the intestinal mucosa [11]. ScFvs isolated from different patients recognized the same tTG epitopes and by ELISA competition experiments we demonstrated that the number of epitopes recognized was restricted to two, distinguished by the ability of the antibodies to recognize mouse tTG [14]. The activities of these in vitro selected antibodies mimics those found in the serum of CD patients, and preliminary work suggests similar biological activity indicating that in vivo studies of these antibodies may provide useful information on the pathogenic roles of these antibodies in CD.

The isolation of disease-specific antibodies, as well as the related gene, is the first step in the generation of animal models through in vivo antibody gene expression. In vivo gene expression has been implemented using a number of different techniques, including injection of naked DNA into the muscle, with or without electroporation [15], coating gold particles with DNA and injecting them with a gene gun [16]. However, expression of recombinant protein is usually transient and only low levels are reached, using these methods. Recently gene transfer systems mediated by vectors based on the adeno-associated virus, have been shown to mediate sustained and prolonged titres of engineered antibody [17-19].

In the present work we describe the production of a series of miniantibody constructs composed of a human autoimmune anti-tTG scFv combined with antibody constant Fc regions from human, rat and mouse. This was sought as a simple approach to rapidly obtain sustained in vivo production of antibodies with specific specificities. In addition to providing appropriate effectors domains, fusion to constant regions also prolongs half life. This represents an innovative tool for the in vivo studies of the pathogenic properties of cloned autoimmune antibody fragments.

## Results

**Figure 1:**
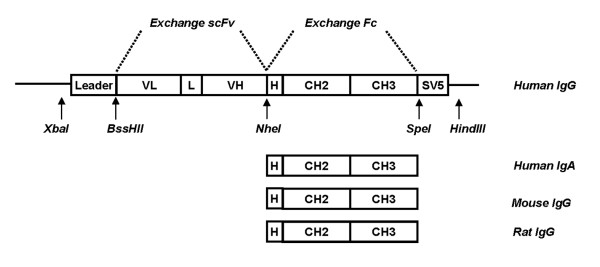
Schematic representation of the cloning vector. The Human IgG1 CH2-CH3 domains gene in the vector pMB-SV5 could be substituted by Human IgA, Mouse and Rat Fc domain genes using restriction sites NheI and SpeI. Different scFvs can be exchanged by using restriction sites BssHII and NheI.

**Figure 2:**
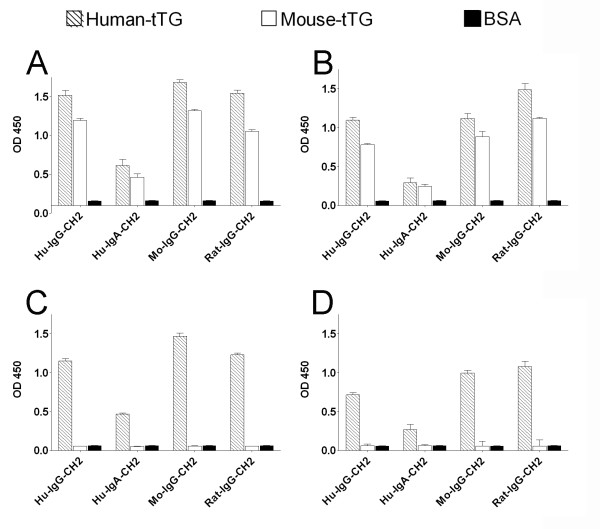
ELISA of supernatants of cultured HEK 293T cells transfected with the series of plasmids pMB-SV5 carrying 2.8 scFv gene (A and B) and 3.7 scFv gene (C and D) fused to CH2-CH3 domains genes from human, mouse and rat. Antigens: human tTG, mouse tTG and BSA. Secondary antibodies: A) and C) biotinylated mAb SV5 and streptavidin conjugated with peroxidase; B) and D) goat anti human, mouse and rat IgG or IgA conjugated with peroxidase.

In a previous paper [11] we described the isolation of human IgA scFvs to tTG from phage antibody libraries obtained from the intestinal lymphocytes of CD patients. Two of these scFvs, indicated as 2.8 tTG, cross-reactive to rodent tTG, and 3.7 tTG, specific for human enzyme, were the reference antibody fragments used to make the series of constructs reported in Fig. 1. The construction of the series pMB-SV5 is reported in Materials and Methods section. It is characterized by a BssHII-NheI cassette for the subcloning of scFvs from the pDAN5 phagemid vector, a NheI-SpeI cassette for the cloning of CH2 and CH3 domains of Fc from different species, and an SV5 tag at the 3' end for uniform detection. Expression of the miniantibodies was driven by a CMV promoter and a leader sequence at the N-terminus was included to allow mammalian secretion. The two selected scFvs (2.8 tTG and 3.7 tTG) were cloned into the 4 different vectors, generating a total of 8 different constructs. All vectors were checked by DNA sequencing and the purified plasmidic DNAs were transfected into HEK 293T cells. The secretion of the miniantibodies in the culture medium was analyzed after 72 h by ELISA on plates coated with either human or mouse tTG. The results, determined by recognition of the SV5 tag at the C-terminus of the constructs (Fig. 2A), or with species specific antibodies (Fig. 2B), for the 2.8 miniantibodies show that they were all able to recognize both antigens with O.D. values ranging from 0.5 (MB-HuA-2.8) to 1.6 (MB-MoG-2.8). Similar results were obtained with the 3.7 miniantibodies, except that recognition was specific for the human enzyme, with no binding to mouse tTG (Fig. 2C and 2D).

Stable cell lines for all the constructs were established by growing transfected cells in the selective agent for hygromicin resistance. Supernatant from individual clones were screened for the best ELISA reactivity and then expanded for further experiments. The average yield of miniantibody production was in the 5–10 mg/liter range using standard culture flask.

**Figure 3:**
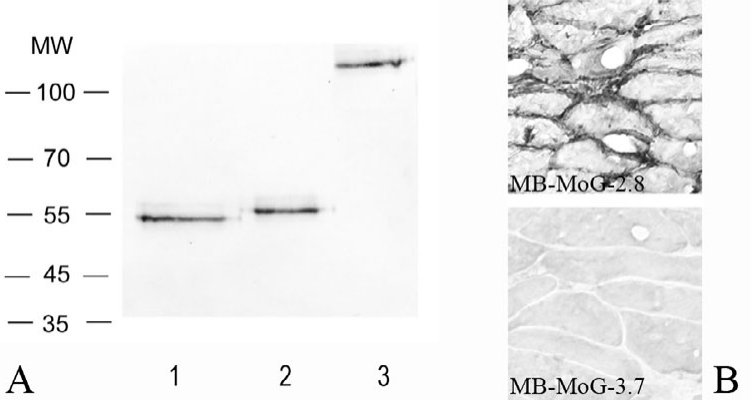
A, Western blotting of the miniantibody MB-MoG-2.8 with reducing agents (lane 2), treated with glycosidase PNGase F (lane 1), and in not reducing not denaturing conditions (lane 3). B, Immunohistochemistry performed on histological section of mouse muscle tissue with the miniantibodies constructs MB-MoG-2.8 and 3.7. Secondary antibodies: biotinylated mAb SV5 followed by streptavidin conjugated with alkaline phosphatase (western blotting) or horseradish peroxidase (immunohistochemistry).

The miniantibodies were analyzed by Western blotting under reducing and non reducing conditions and after treatment of the purified miniantibodies with glycosidase PNGaseF to assay the level of glycosylation. The results for the miniantibody MB-MoG-2.8 are reported, as an example, in Fig. 3A. The predicted molecular weight of the miniantibodies, result of the fusion of the scFv with constant domains and SV5 tag, is about 55 kDa.

We found bands of the predicted molecular weight in the samples treated with reducing agent and a slight increase in the electrophoretic mobility in the deglycosylated samples, indicating that the miniantibodies are glycosylated in HEK 239T cells. Under non reducing, non denaturing conditions, a high molecular weight band, explained by the interchain disulfide bond in the Hinge region, was obtained.

**Figure 4:**
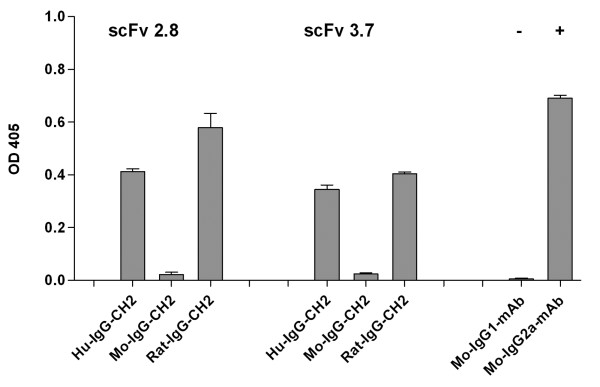
Complement fixation assay with the miniantibodies constructs MB-HuG-2.8 and 3.7, MB-MoG-2.8 and 3.7, MB-RaG-2.8 and 3.7. The binding of C1q to the miniantibodies is revealed with biotinylated anti-C1q and streptavidin conjugated with alkaline phosphatase. Positive and negative control are represented by the murine anti-His D8 (IgG2a) and CUB7402 (IgG1) mAb, both recognizing the coated tTG.

The ability of the miniantibodies to activate the classic complement pathway was tested with an in vitro assay, as reported in Fig. 4, in which C1q deposition was measured using an ELISA assay. The results showed that human and rat miniantibodies, of IgG1 and IgG2b origin respectively, were able to bind C1q, the first component of the classic complement cascade, as described in the literature [20,21], whereas the mouse miniantibody of IgG1 origin could not bind C1q [22].

**Figure 5:**
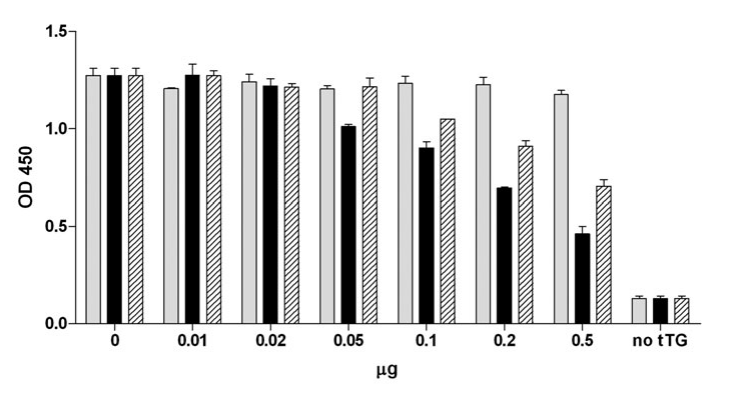
Inhibitory effect of purified MB-MoG-3.7 (grey bars), MB-MoG-2.8 (black bars) and mAb CUB7402 (hatched bars) on mouse tTG activity. Elisa plates coated with gliadin, a tTG substrate, are incubated with 0.2 mM 5-(biotinamido)pentylamine and 0.25 μg of mouse tTG, with increasing amounts of purified miniantibody or mAb. The incorporation of 5-(biotinamido)pentylamine is revealed by streptavidin conjugated with peroxidase.

The miniantibodies were analyzed for their ability to recognize tTG on histological sections. These experiments were undertaken in view of the possible use of these miniantibodies in in vivo studies. The immunolabeling of histological sections of mouse muscle is shown in Fig. 3B, using purified MB-MoG-2.8 and 3.7. Staining was only detected for MB-MoG-2.8 with specific recognition of the extracellular tTG present at the muscular endomysium and perimysium. The same pattern was found when miniantibodies with the human or rat Fc were used (not shown). For the same reasons, an assay on the inhibition of tTG activity by miniantibodies was tested. We have already shown that scFvs to tTG isolated from the intestinal lymphocytes of CD patients inhibit the in vitro transamidation activity of tTG [23]. Fig. 5 shows that the coupling of 5-(biotinamido)pentylamine to gliadin (a tTG substrate) is catalyzed by mouse purified recombinant tTG, which is inhibited by miniantibody MB-MoG-2.8, but not miniantibody MB-MoG-3.7, confirming the specificity observed by ELISA. The inhibition closely mirrors the values previously described for the scFv [23] in a similar assay. In comparing the two miniantibodies with the commercial tTG-specific monoclonal antibody CUB7402, the results show that the percentage of enzyme activity inhibition is higher with the miniantibodies compared to CUB7402, using the same enzyme to antibody ratio. We believe that this is due to both a difference in affinity and the different epitope recognized by the CD derived antibodies.

**Figure 6:**
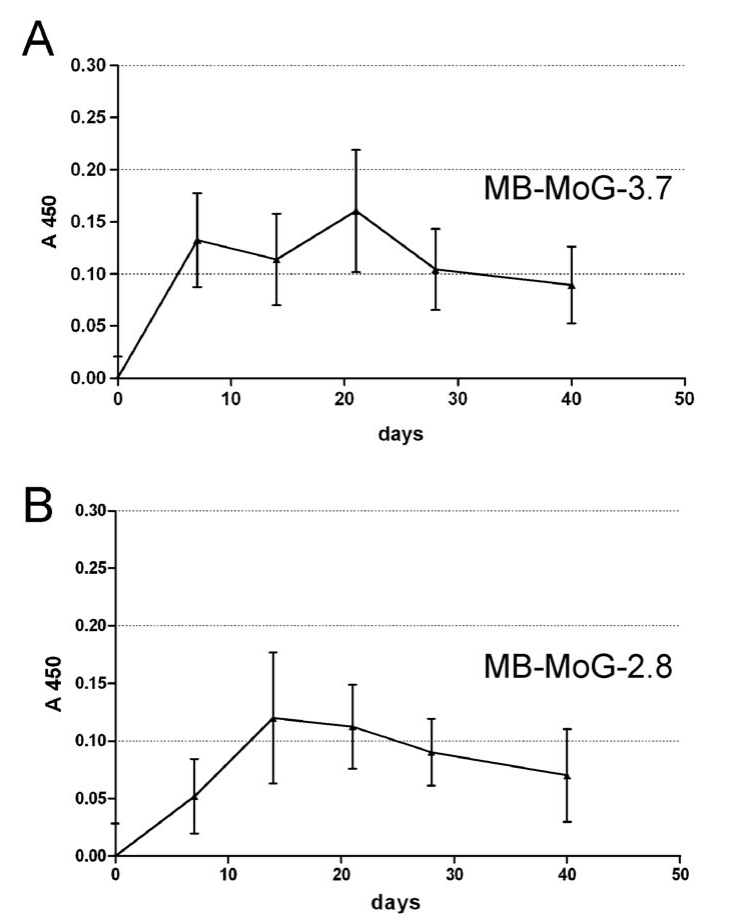
ELISA time course of the serum anti-tTG miniantibody average titer in 8 BALB/c mice injected at 0 and 14 days with pMB-MoG-3.7 (panel A) and 2.8 (panel B) DNA. Serum dilution 1:50. Secondary antibodies: biotinylated mAb SV5 and streptavidin conjugated with peroxidase.

**Figure 7:**
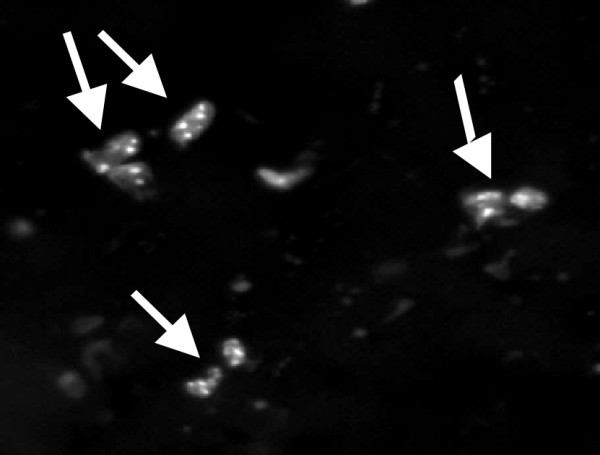
In situ PCR on histological section of quadriceps muscle of a mouse injected with pMB-MoG-3.7 construct. PCR was performed after 40 days since injection. The arrows point at cells with positive reaction.

The in vivo expression of selected miniantibodies was studied by using DNA vaccination protocols. According to this method of gene transfer, DNA is delivered directly to the muscle of the animal where it is internalized by the muscular fibers and expressed if an appropriate eukaryotic promoter is present. In our case, in order to evaluate the possibility of using miniantibodies for in vivo studies of the biological activities of autoimmune antibodies, we used an anti-tTG scFv that recognizes mouse tTG, and a second construct which did not cross-react with rodent tTG. In the latter case, the purpose was to monitor the serum level of an antibody not sequestered by tTG at the tissue level, which may occur, if an antibody recognizing mouse tTG is used. 50 μg of purified pMB-MoG-2.8 and pMB-MoG-3.7 DNA were injected twice into the quadriceps of eight BALB/c mice at an interval of 14 days. The mice were periodically examined for the presence of reactive miniantibodies to tTG at serum levels by ELISA. As outlined in Fig. 6, for both constructs low but detectable levels of miniantibodies to tTG were measured in the blood samples taken up to 40 days after the injection. Mouse sera were also investigated for the possible presence of antibodies raised against the miniantibody molecule. This was done by ELISA, adsorbing purified MB-MoG-2.8 and MB-MoG-3.7 on plastic wells. No evidence of an induced immune response was found, with sera from the 2.8 or 3.7 treated mice all negative at 40 days (data not shown). At the end of the experiments the mice were sacrificed and the hindquarter muscle examined by in situ PCR for the presence of miniantibodies DNA. The result, depicted in Fig. 7, showed a positive labeling for scattered muscular fibers, indicating the continued presence of the plasmid DNA.

## Discussion

Engineered antibodies are increasingly being used as therapeutic agents in numerous cases including oncology, autoimmunity, inflammation and infectious diseases [24]. Combinatorial approaches have been applied to scFvs isolated from phage display libraries, modifying the reactive V regions by fusion with a range of molecules to improve the antibody stability and avidity [25,26], to alter the effector functions [27,28], to balance the pharmacokinetics [29], to facilitate the purification [30], or to combine different antibodies giving rise to bifunctional antibodies [31-33]. In the case of the present work, the fusion of human autoimmune scFvs to the Fc domains of different species had the goal of using such constructs for expression in vivo and possibly generate an autoimmune animal model. Such direct scFv-Fc fusions have been widely used and expressed in yeast [34,35] and mammalian cells [36,37], with activity similar to full length IgGs in most assays, with the advantages of dimerization and effector functions, provided by the fused Fc domain. The most critical passage of this approach was the preservation of the antibody reactivity after fusion with Fc domains from other species. This was implemented by the creation of a novel mammalian expression vector in which either scFv or Fc domain could be easily switched using compatible restriction sites. As demonstrated by ELISA, all the chimeric constructs recognized the tTG antigen, and western blotting showed the expected higher molecular weight bands corresponding to the dimeric form. The binding activity of the constructs with the scFv 2.8, crossreactive to rodent tTG, was also preserved to mouse tTG tested in ELISA as well as in an immunochemical assay on histological section of mouse muscle, as was the ability to inhibit the crosslinking activity of tTG. Complement activation measured by C1q deposition was confirmed for the human and rat miniantibody constructs reflecting the presence of the CH2 domain and correct glycosylation produced by the transfected 293T cell.

Injection of plasmid DNA was used to induce in vivo expression of miniantibodies. This approach derives from extensive studies on DNA vaccination in which naked plasmid DNA, coding for an antigenic protein, is transfected into muscle cells in vivo either by injection [38,39] or using a "gene gun" [40]. This results in expression of the vector-encoded antigen, which induces cellular and humoral responses [41]. In a similar vein, injection of DNA coding for an anti-tumoral scFv has also been carried out [42]. Although other studies have demonstrated both cellular and humoral responses against human scFvs in mice [43] with a reduction of the therapeutic potential, this feature can also be exploited to generate anti-idiotypic responses against scFvs derived from mouse lymphomas [44,45].

In these experiments, the effectiveness of the anti-idiotypic response was enhanced by fusing the cloned antibody expressed by the tumoral cells to an additional CH3 antigenic region, as originally suggested by Syrengelas et al. [46]. In the present study, since a human scFv xenogenic for mice was used, we sought to minimize the host immune response by using the scFv fused to a CH2-CH3 syngenic mouse Fc region. The outcomes of the experiments have confirmed the validity of this approach, with a detectable production of miniantibodies in the serum for at least 40 days and a peak of production after 20 days.

## Conclusion

The reactivity of the serum miniantibodies in ELISA together with the apparent lack of humoral response against the miniantibody molecule, led us to conclude that the human scFv-mouse Fc fusion miniantibodies are poorly immunogenic in the mouse under the experimental conditions used here. In conclusion, our results indicate that chimeric proteins generated by fusion of human scFvs to human, murine and rat Fc regions are effectively produced and secreted by cultured cells; the polypeptides dimerize, forming disulfide bridges, so increasing the valency of the miniantibody; the miniantibodies retain the antigen recognition both in ELISA and immunohistology and the ability to activate complement. The inhibitory properties of the scFv are preserved and, upon intramuscular injection of the plasmid, the ELISA antibody titre is still detectable after 40 days, suggesting the absence of an immune response by the host when a syngenic Fc fragment is present in the construct. While this indicates that the approach is functional and will be useful in the in vivo study of the role of autoimmune antibodies, in the absence of complicating immunological factors, the period of expression obtained here is relatively short when compared to the long time scale involved in most autoimmune diseases, which occurs over a period of years. For this reason it may be appropriate to explore alternative in vivo expression methods, such as adeno-associated virus vector, which has been shown to induce expression over a prolonged period.

## Methods

### Bacterial strains and enzymes

*DH5aF' *(F'/endA1 hsdR17 (rK^- ^mK^+^) supE44 thi-1 recA1 gyrA (Nalr) relA1 D (lacZYA-argF)U169 deoR (F80dlacD(lacZ)M15)) strain was used for the cloning of pDAN5, pMB-SV5 and derivates, pCDNA3.1/Hygro(+) and pTrcHisB. Molecular biology enzymes were purchased from New England Biolabs, Promega or Life Technologies.

### Antigens

Human tTG gene was cloned in pTrcHisB as described [47]. Mouse tTG gene was cloned in pTrcHisB as described [48]. Protein purification was performed as described in [14].

### RNA extraction and cDNA synthesis

Peripheral blood lymphocytes from a healthy donor and spleen lymphocytes from mouse and rat were separated by density gradient centrifugation on Ficoll Hypaque (Pharmacia). Total RNA was then prepared as described [49]. cDNA was prepared using SuperScript II Reverse Transcriptase (Gibco BRL) with random hexamers.

### pCDNA3.1/Hygro(+) modification

pCDNA3.1/Hygro(+) plasmid vector (Invitrogen) was modified as follows: NheI restriction site was exchanged with XbaI, PmeI site was exchanged with HindIII site by inverse polymerase chain reaction (PCR) using the primers Hygro-XbaI-INV antisense and Hygro-HindIII-INV sense; the primers are reported as A and B in Table 1. ScFv 2.8 [11] was PCR amplified from pDAN plasmid vector by successive amplification with sense primers 1-XbaI-leader, 2-leader-intron and 3-BssHII-scFv (Table 1, primers 1, 2 and 3), which introduce the XbaI site, a secretion leader and a mini-intron as described in [50] and the BssHII site at the 5' end, and antisense primer scFv-NheI-HindIII (Table 1, primer 4), which introduces the NheI and HindIII sites at the 3' end. PCR fragment was cloned as XbaI – HindIII in the modified pCDNA3.1/Hygro(+) vector.

**Table 1:** Primers. List of oligonucleotides used for vector construction and Fc region cloning.

Number	Name	Orientation	Sequence
A	Hygro-XbaI-inv	Antisense	AGCTTCTAGACAGCTTGGGTCTCCCTATAG
B	Hygro-HindIII-inv	Sense	AGCTAAGCTTAAACCCGCTGATCAGC
1	1-XbaI-leader	Sense	CAGGCGTCTAGATGCCACCATGGGCTGGAGCCTGATCCTCCTGTTCCTCGTCGCTGTGGCTACAGGTAAGGG
2	2-leader-intron	Sense	TGTGGCTACAGGTAAGGGGCTCACAGTAGCAGGCTTGAGGTCTGGACATATATATGGGTGACAATGACATCCAC
3	3-BssHII-scFv	Sense	GGTGACAATGACATCCACTTTGCCTTTCTCTCCACAGGTGGCGCGCATGCCGACATCCGGTTGACCCAG
4	scFv-NheI-HindIII	Antisense	CCGCTAAGCTTCGCCTGGCTAGCAAAAGCGTCCGTCGTATC
5	HuGCH2-s	Sense	AGGCGGCTAGCGACAAAACTCACACATGCCCACCGTGCCCA
6	HuGCH3-SV5-a	Antisense	CTGCTAAGCTTTTAAGTACTATCCAGGCCCAGCAGTGGGTTTGGGATTGGTTTGCCACTAGTTTTACCCGGGGACAGGGAGAG
7	HuACH2-s	Sense	CAGGCGGCT AGCGTTCCCTCAACTCCACCTACC
8	HuACH3-a	Antisense	CCGCTACTAGTTTTACCCGCCAAGCGGTCGAT
9	MoGCH3-a	Antisense	CCGCTACTAGTTTTACCAGGAGAGTGGGAGAG
10	MoGCH2-s	Sense	CAGGCG GCT AGC GGTTGTAAGCCTTGCATATGTACA
11	RaGCH3-a	Antisense	CCGCTACTAGTTTTACCCGGAGGCCGGGAGATG
12	RaGCH2-s	Sense	CAGGCG GCT AGC CACAAATGCCCTACATGCCCT

### Cloning of Human Fc gene

The human IgG1 CH2 and CH3 domains gene was amplified from lymphocyte cDNA by using the primer sense HuGCH2-s and antisense HuGCH3-SV5-a (Table 1, primers 5 and 6), which introduce SpeI site and the SV5 tag sequence for mAb recognition [51] at the 3' end. PCR fragment was cloned as NheI-HindIII into the pCDNA3.1/Hygro(+) vector modified as described and carrying the scFv 2.8; the resulting vector was named pMB-HuG-2.8. The series of vector obtained by exchange of different scFv and Fc was called pMB-SV5.

### Exchange of constant domains and scFvs

The set of oligonucleotide primers for amplification of Fc domain genes was designed to comprise the CH2-CH3 domains including the flexible hinge region. The CH2-CH3 domain genes were PCR amplified by using the sense primer HuACH2-s and antisense HuACH3-a for human IgA, MoGCH2-s and MoGCH3-a for mouse IgG1, RaGCH2-s and RaGCH3-a for rat IgG2b. All primers are reported in Table 1. The PCR fragments were cloned in pMB-HuG-2.8 vector replacing the resident Fc domain gene by cutting with NheI and SpeI and ligation.

The cloning of 3.7 scFv gene was performed by extraction of scFv gene from phagemid pDAN5 clone by cutting with BssHII and NheI and direct cloning in the series of vectors pMB-SV5 cut with the same enzymes.

### HEK 293T transfection and selection

The human kidney derived HEK 293T cell line was cultured in D-MEM medium (GIBCO) supplemented with 10% fetal calf serum (FCS). Cells were harvested by shaking and plated in a 24 well microtiter plate (2 × 10^5 ^cells per well). For transient transfection, after 24 h, 1 μg of purified plasmid DNA resuspended in 50 μl of D-MEM without FCS and 2 μl of Lipofectamine 2000 (Invitrogen) in 50 μl of D-MEM were mixed, left at RT for 20 min and added to each well of cultured cells. The cells were grown for further 24/48 h and the supernatant inspected for miniantibody production. Stable cell clones secreting miniantibodies were obtained by treating the cells in the same way as for the transient transfection, diluting the cells 1:10 with fresh medium after 24 h from transfection and adding 400 μg/ml of antibiotic Hygromicin (Invitrogen) for the selection of resistant cells. After 10 days of culture, the Hygromicin concentration was reduced to 200 μg/ml.

### ELISA

ELISA was performed by coating ELISA plates with purified human or mouse recombinant tTG at 10 μg/ml diluted in phosphate-buffered saline (PBS) for 15 h at 4°C. Wells were blocked with 2% non-fat milk in PBS (MPBS). The primary antibodies were the supernatants of cultured HEK 293T cells diluted 1:5 with 2.5% MPBS or sera of mice injected with plasmid DNA diluted 1:50 with 2% MPBS. Secondary antibodies used were biotinylated mAb SV5 [51] recognizing the SV5 tag found at the miniantibody C-terminus and goat anti human, mouse and rat IgG or IgA conjugated with peroxidase. The secondary antibodies were used as following: a) biotinylated mAb SV5 diluted 1:2000 with 2% MPBS, followed by streptavidine conjugated with horseradish-peroxidase (HRP) (Pierce) diluted 1:2000, b) goat anti human, mouse and rat IgG or IgA conjugated with peroxidase (Dako) diluted 1:1000. Each step was followed by three washes with PBS plus 0,1% Tween20 (PBST) and three washes with PBS. All the immunocomplexes were revealed with tetramethyl-benzidine (TMB) and read at O.D._450_.

### Serum-free cell cultures and Protein G purification

Serum-free supernatants for protein-G purification were obtained as following: stable cell clones grown at confluence in 25 cm^2 ^flasks were harvested, centrifuged at 1200 × g and resuspended in FCS-free D-MEM medium. Cells were allowed to grow for further 48 h and the supernatants collected.

Miniantibodies produced in FCS-free cell culture supernatants were purified by using a HiTrap protein G column (GE Healthcare) following standard procedures. Briefly, 50 ml of serum-free culture were passed through the protein G column; the column was washed with 20 ml of 100 mM Tris-HCl, pH 8.0, and 20 ml of 10 mM Tris-HCl, pH 8.0. Purified miniantibodies were eluted with 50 mM Glycine, pH 3.0, and immediately buffered with Tris-HCl, pH 8.0.

### tTG inhibition assay

ELISA plate wells were adsorbed with 20 μg/ml purified gliadin for 2 h at 37°C and washed twice with PBS. To each well 100 μl of a solution of 5-(biotinamido)pentylamine (Pierce) 0.2 mM, 0.25 μg of purified mouse tTG in NaCl 150 mM, Tris 50 mM pH 7.5 with increasing amount of either protein G purified miniantibody or commercial tTG-specific monoclonal antibody CUB7402 (Bio Optica, Milan), ranging from 0 to 0.5 μg per microwell, were added. After 1 h incubation at 37°C, the wells were washed three times with PBS plus 1% Tween20 and three times with PBS. 100 μl of a solution of streptavidin conjugated with alkaline phosphatase (Pierce) 1:2000 in PBS 2% bovine serum albumin (BSA) were added to each well and incubated for 1 h at RT. After extensive washing, the tTG activity, based on coupling of 5-(biotinamido)pentylamine to gliadin by tTG, was revealed by adding 100 μl of 4-nitrophenyl phosphate (pNPP) (Sigma) and read at O.D._405_.

### Complement fixation assay

The complement fixation assay was performed by coating ELISA plates with recombinant human tTG at 100 μg/ml for 15 h at 4°C. Wells were blocked with MPBS for 1 h and then primary antibodies were incubated for 1 hr at RT. Protein G purified miniantibodies were used as primary antibodies, at a concentration of 0,5 μg per microwell in 2% MPBS. An anti-histidine tag murine monoclonal antibody of IgG2a isotype His-probe D8 (Santa Cruz) and a commercial anti-tTG murine IgG1 CUB7402 (Neomarker), both recognizing the coated tTG, were used diluted 1:500 in MPBS as a positive and negative control, respectively. After three washes with PBST and three with PBS, purified human complement component C1q (Quidel) 3 μg/ml in 0,1% MPBS with 0,05% Tween20 was incubated for 1 hour. Following washes a biotin-labelled anti-C1q antibody (Quidel) diluted 1:3000 in the same buffer of C1q was incubated for 1 hour, followed by alkaline phosphatase (AP)-conjugated streptavidin (Pierce) 1:3000 in PBS with BSA 2% for 45 minutes. Reaction was revealed with 4-nitrophenyl phosphate (pNPP) (Sigma) and read at O.D._405_.

### Western blotting

Sodium dodecyl sulphate polyacrylamide gel electrophoresis (SDS PAGE) under reducing conditions was performed according to standard techniques. To perform SDS PAGE under non-reducing conditions, proteins were loaded in sample buffer without β-mercaptoethanol. To assess glycosylation, samples were treated with or without deglycosylase PNGaseF (New England Biolabs) according to the manufacturer instructions. Cell culture supernatants containing miniantibody fractions were separated by SDS PAGE and transferred onto nitrocellulose (Amersham) by semi dry blotting using the Pharmacia Multiphor II. The membrane was blocked using 2% MPBS for 1 hour at room temperature. Biotinylated mAb SV5 was used as primary antibody. After 2 h incubation at RT and extensive washing with PBS plus 0.1% Tween20, the nitrocellulose was subsequently incubated with alkaline phospatase conjugated streptavidin (Pierce) diluted 1:1000 and revealed by the chromogenic substrate BCIP and NBT.

### Immunohistochemistry

Immunoperoxidase staining was performed on histological sections of mouse muscle prepared according to standard techniques. Miniantibodies from HEK 293T culture supernatant were added to the sections, incubated for 30' at room temperature in a moist chamber, followed by biotinylated mAb SV5 diluted 1:1500 and peroxidase conjugated Streptavidin (Pierce) diluted 1:2500 and diaminobenzidine (DAB) as substrate.

### DNA vaccination

Eight healthy, 8 week-old, female, BALB/cAnNHsd mice were purchased from Harlan Italy. Mice were injected with 50 μl of bupivacaine 0.50% in isotonic NaCl into the quadriceps muscle. Five days later, the bupivacaine treated zones were injected with 50 μg of purified pMB-MoG-2.8 and pMB-MoG-3.7 plasmidic DNA in 50 μl PBS. A second injection with the same DNA quantity was made after 14 days. Small volumes of blood were periodically sampled from the mandibular artery and analyzed for the presence of serum miniantibodies. Animal care and treatment were conducted in conformity with institutional guidelines in compliance with national and international laws and policies (European Economic Community [EEC] Council Directive 86/609; OJL 358; December 12, 1987).

### In situ PCR

Frozen mouse quadriceps muscle tissue histological sections (5–10 μm) fixed on SuperFrost slides, were rehydrated to nuclease-free water through graded fresh aqueous solution of ethanol (100%, 90%, 80%) then permeabilized in a 0.01% Triton-X 100/PBS solution for 2 min, and rinsed in PBS for 2 min. Primers VLPTL and VHPT2 [3] and 5 mM dUTP Cy3fluorescent nucleotides (Amersham Pharmacia) were used for direct labeling of the amplicon. The direct fluorescent in situ PCR was performed using the following cycle: 94°C, 30 s; 53°C, 60 s; 72°C, 60 s, repeated 15 times. After the PCR reaction the slides were washed twice with PBS for 5 min and then counter-stained with 4',6-Diamino-2-phenylindole (DAPI) (Vectashield, Burlingame CA) and directly observed under a fluorescent microscope (Olympus Optical, Shinjuku-ku, Tokyo, Japan).

### Detection of anti-idiotype response

ELISA plates were coated with purified MB-MoG-2.8 and MB-MoG-3.7, at 10 ug/ml for 15 h at 4°C. Wells were blocked with 2% MBPS and incubated with sera of mice diluted 1:50 with 2% MPBS, for 2 h at 37°C. After extensive washing with PBST and PBS, secondary antibody anti-mouse Fab-specific conjugated with HRP (Jackson Immunoresearch) diluted 1:5000 with MPBS was added and incubated for 1 h at 37°C. All the immunocomplexes were revealed with TMB (Sigma), the reaction was stopped with 1 M sulphuric acid and read at OD_450_.

## Authors' contributions

RM, DS, OB and MB conceived, designed, and coordinated the original project and provided scientific and administrative support. RD, FZ, PS and FF performed the construction of the vector series pMB-SV5, transfection and maintaining of cell cultures, ELISAs, western blotting, tTG inhibition assay and immunohistochemistry. MS performed the in vivo injection of DNA constructs. SC performed the in situ PCR. DS and ARMB wrote and revised the manuscript. All authors read and approved the final manuscript.
